# Sexual shape dimorphism accelerated by male–male competition, but not prevented by sex-indiscriminate parental care in dung beetles (Scarabaeidae)

**DOI:** 10.1002/ece3.1558

**Published:** 2015-06-19

**Authors:** Shigeki Kishi, Koh-Ichi Takakura, Takayoshi Nishida

**Affiliations:** 1 Center for Ecological Research, Kyoto UniversityShiga, Japan; 2 Department of Biological Resource, School of Environmental Science, The University of Shiga PrefectureShiga, Japan; 3 Department of Ecosystem Studies, School of Environmental Science, The University of Shiga PrefectureShiga, Japan

**Keywords:** Dung beetle, parental investment, sexual dimorphism, sexual size dimorphism

## Abstract

Dimorphic sexual differences in shape and body size are called sexual dimorphism and sexual size dimorphism, respectively. The degrees of both dimorphisms are considered to increase with sexual selection, represented by male–male competition. However, the degrees of the two dimorphisms often differ within a species. In some dung beetles, typical sexual shape dimorphisms are seen in male horns and other exaggerated traits, although sexual size dimorphism looks rare. We hypothesized that the evolution of this sexual shape dimorphism without sexual size dimorphism is caused by male–male competition and their crucial and sex-indiscriminate provisioning behaviors, in which parents provide the equivalent size of brood ball with each of both sons and daughters indiscriminately. As a result of individual-based model simulations, we show that parents evolve to provide each of sons and daughters with the optimal amount of resource for a son when parents do not distinguish the sex of offspring and males compete for mates. This result explains why crucial and sex-indiscriminate parental provisioning does not prevent the evolution of sexual shape dimorphism. The model result was supported by empirical data of Scarabaeidae beetles. In some dung beetles, sexual size dimorphism is absent, compared with significant sexual size dimorphism in other horned beetles, although both groups exhibit similar degrees of sexual shape dimorphism in male horns and other exaggerated traits.

## Introduction

Dimorphic sexual differences in character size and shape are called sexual size dimorphism (SSD) (Andersson 1994) and sexual dimorphism (Emlen and Nijhout 2000), respectively. The degrees of SSD and sexual dimorphism in shape have been considered to increase with intense sexual and natural selection (Székely et al. 2000; Isaac 2005; Fairbairn et al. 2007). Male–male competition is a typical mechanism of sexual selection, often making larger males with larger horns advantageous in the reproductive success (Eberhard 1980). However, the degrees of SSD and sexual shape dimorphism often greatly differ within a species, because evolutionary and developmental factors differently affect them (Badyaev 2002). For example, the degree of SSD does not simply correlate to relative male horn length to body size in coleopteran beetles (Kawano 2006), probably because the degree of SSD and relative male horn length are determined by different life-historical traits. However, among those traits that affect SSD and/or sexual shape dimorphism, it is little understood to which extent each factor affects the two dimorphisms (Fairbairn 1997; Blanckenhorn 2005).

If parents indiscriminately feed sons and daughters, the degree of SSD should be smaller (Badyaev 2002). On the other hand, if parents distinguish the sex of offspring and differently provision sons and daughters, SSD should occur in adult body size of sons and daughters in response to the difference in the amount of food and cares. For example, fathers of the budgerigar, *Melopsittacus undulatus*, provide more food for female-biased broods than for male-biased broods (Stamps et al. 1987), although the differential food provisioning may be caused by differential food requirements of sons and daughters (Anderson et al. 1993). However, parents of many organisms do not appear to distinguish between offspring sexes in the amount of food, and their sons and daughters do not appear to differentially beg parents to provide more food. For example, in some dung beetles, leaf-rolling beetles, bark beetles, and burying beetles, parents provide crucial amount of food for both sexes of offspring indiscriminately (Kurosawa et al. 1996). In those beetles, SSD looks rare probably due to the indiscriminate and crucial food provisioning for sons and daughters, while significant sexual shape dimorphism has been reported in those dung beetles and leaf-rolling beetles (Emlen et al. 2005; Kawano 2006).

While crucial and sex-indiscriminate provisioning would prevent SSD, it is unclear whether it also prevents the evolution of sexual shape dimorphism, or not. In a species that larger males develop larger sexual ornaments and are likely to win the male–male combat for mates, the optimal amount of food for a son is expected to be larger than that for a daughter (Clutton-Brock 1991). In this situation, if parents do not distinguish the sex of offspring and then indiscriminately provide each of sons and daughters with less than the optimal amount of food for a son, the body size and the sexual ornament size of males should be limited. Hunt and Simmons (2004) argued that parents of a dung beetle, *Onthophagus taurus*, may provide the intermediate amount of food between the optimum for a son and the optimum for a daughter. This argument suggests that parental provisioning should not only limit the body size but also horn size of males and then prevent the evolution of sexual shape dimorphism. On the other hand, Kishi and Nishida (2008) showed that parents of a dung beetle, *O. atripennis*, provide the optimal amount of food for a son for each of both sons and daughters. This study suggests that sex-indiscriminate parental provisioning should not limit the male body size and sexual ornaments and then should not prevent the evolution of sexual shape dimorphism.

To examine whether crucial and sex-indiscriminate parental provisioning prevents the evolution of sexual shape dimorphism or not, we used two different approaches: simulation models and empirical data. At first, we built individual-based simulation models, in which parents determine the adult body size of offspring depending on the virtual genotype. We compared a simulation result when parents distinguish the sex of offspring with another result when parents do not. If the evolutionary consequence of the amount of food provided by the sex-indiscriminate parents results in the optimum for a son when male–male competition occurs, it suggests that crucial and sex-indiscriminate parental provisioning does not prevent the evolution of sexual shape dimorphism, though limiting SSD. Second, we measured and compared the degrees of the two dimorphisms between Japanese dung beetles and other horned beetles without crucial parental provisioning in the same family, Scarabaeidae. If crucial and sex-indiscriminate parental provisioning limits SSD but not the evolution of sexual shape dimorphism, the degree of SSD is rarer in dung beetles but the degree of relative horn length does not differ between dung beetles and other horned beetles.

## Materials and Methods

### Model

To investigate how much amount of food resource per offspring parents evolve to provide, we built four individual-based models by combining two alternative conditions. One condition was the distinction of offspring sex, whether parents distinguished the sex of offspring and differently invested in a son and a daughter (D) or not (d). The other condition was the occurrence of male–male competition, whether males competed for mates (C) or not (c). Each of four models is then called DC, Dc, dC, and dc, respectively. For example, the model DC was assumed that parents distinguished between sons and daughters and fed them differently, and males competed for mates. We compared the evolutionarily resultant amount of parental resource provisioning per offspring between the four models. In all of the models, the model organisms were assumed as diploidy and they reproduced sexually with discrete generations. Each trait of the model organism was considered as quantitative and governed by a polygene. The life history consisted mainly of three phases: mating, reproduction, and density regulation.

We used up to three quantitative traits involved with the resource allocation to offspring: the investment sex ratio, the amount of resource per son and that per daughter. In models DC and Dc, in which parents distinguish the sex of offspring, we incorporated all of three traits to the model. In models dC and dc, in which parents do not distinguish the sex of offspring, we incorporated two traits, because resource amounts provided per son and per daughter were assumed to be controlled by a single quantitative trait. We assumed that each trait was governed by 100 independent loci, 50 on the chromosomes derived from the mother and the other 50 on those from the father. Each locus was either dominant or recessive. The effect of each allele on the phenotype was considered as completely additive. The phenotype of the investment sex ratio was expressed on a linear scale ranging from zero to 1.0, and it was calculated as the proportion of dominant alleles in the 100 loci. The phenotypes of the amount of parental resource provisioning for a son and that for a daughter were calculated as 10 times the proportion of dominant alleles at the 100 loci, and therefore, they range from 0.0 to 10.0. Mutation rate of each allele was set to 0.00001. Each locus was completely independent of others. The phenotypic values of investment sex ratio and the amount of resource provided for a son and for a daughter were assumed to be expressed as means of the mother’s and father’s trait values, because in most species of dung beetle, parents cooperatively construct brood balls (Halffter 1997). We also simulated other versions that each mother exclusively determined the amount of resource for a son and for a daughter according to her trait values, but found that the simulation results were quite similar with the results of this study.

We started model simulations with the mating phase, followed by reproduction and density regulation stages in a generation. We let each female mate once with a male during her life. At first, each female randomly chose *n* males as candidate mates from the population. Then, in the models DC and dC with male–male competition, the female was assumed to mate with the largest male among them, while in the models Dc and dc without male–male competition, the female was assumed to mate with a male randomly selected among them. The number of candidate males *n* ranged depending on a Poisson distribution with the expected value of 3.0. After this mating process, male candidates were returned to the population and hence might be repeatedly selected as candidates.

**Figure 1:**
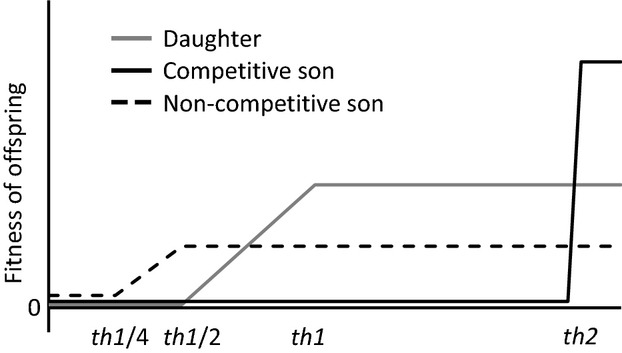
Fitness curves in relation to the body size (*x*-axis) assumed in the present study. The gray, black, and dashed lines indicate the fitness curves of daughters, competitive sons, and noncompetitive sons, respectively. Between the minimum *th1*/4 and the maximum body size *th2*, the number of eggs per female linearly increases from *th1*/2 to *th1*.

After the mating phase, the reproduction phase followed. The total amount of resource that a female used for reproduction during her life was determined by the female body size. Every female larger than a threshold value *th*_1_ got a maximum amount of resource *R*_max_. However when females were smaller than the threshold value, the total amount of resource linearly reduced with the female’s body size, finally reaching zero at the female body size *th*_1_/2 (Fig.1). We set the threshold *th*_1_ and the total amount of resource *R*_max_ to 2.0 and 30.0, respectively. In preliminary simulations, we confirmed that the threshold body size was the optimal body size for a daughter, because of the best efficiency of fitness return per investment. The genotype of offspring was determined by those of parents. We assumed that each produced egg was made from two haploid gametes each from the mother and the father because the model organism was diploidy. The gamete had a set of alleles coding polygenetic traits, which were randomly chosen from either one of homologous loci.

In the reproduction phase, resource allocation to each offspring consists of two steps: sex allocation and following individual allocation. First, parents divide their resource into two batches, one for daughters and the other for sons, according to the mean phenotypic value of the investment sex ratio. Then, using the batch for daughters, parents repeatedly produced daughters until the remaining resource decreased to less than the amount allocated for a daughter. The amount of resource for an individual daughter was the mean value of the two genotypes from parents, as described above. Then, parents produced sons in similar way. On the other hand, in the models dC and dc, without distinction of the sex of their offspring, parents were assumed to give the equivalent amount of food for each of sons and daughters.

The amount of food given to an offspring directly determined the adult body size, but the body size ranged from the lower threshold *th*_1_/4 to the upper ceiling *th*_2_, corresponding to physical and physiological constraints (Smith and Fretwell 1974; Hunt and Simmons 2004; Kishi and Nishida 2008). The survival rate of an individual with the lower threshold body size (i.e., 0.5) was set to 0.0 and that of an individual with more than the lower threshold linearly increased with the body size to *th*_1_/2 (i.e., 1.0) where the survival rate was set to 1.0. The upper threshold *th*_2_ was set to 4.0. Therefore, the male and female body sizes at the mating and investing phases are congruent with the amount of food that parents provide for a son and for a daughter, respectively.

At the last phase in a generation, we set the density regulation phase to avoid a population explosion. In this phase, an individual was randomly selected from the population and repeatedly removed until the population size was equal to or smaller than the carrying capacity *K*, which we set 1000 individuals in all of the models. Hence, the density regulation process did not affect the evolution of traits.

We set initial values as follows: The initial population comprised 500 females and 500 males. The mean investment sex ratio was set to 0.5, and the mean amount of resource provided to a single offspring was set to 2.0. On the basis of these values, the allele at each locus was randomly determined.

By running preliminary simulations, we confirmed that the mean body size reached an equilibrium within 500 generations and also that the simulation outcomes were merely affected by the initial mean values of investment in an offspring and body size. We then conducted 100 simulation runs for 1000 generations in each model and then compared the amount of resource provided to a daughter and to a son between the models. Furthermore, to confirm reproductive success of each individual with a given body size in each model, we drew fitness curves for males and females in the resultant population after 1000 generations in the following way. We put an individual with a given body size into the resultant population and ran the simulation for only one generation and counted the number of offspring that the individual left for next generation. This process was carried out for 3000 individuals of each sex with various body sizes.

### Specimen measurements

Crucial and sex-indiscriminate parental provisioning is known in dung beetle species (subfamily, Scarabaeidae). A beetle mother constructs brood balls composed of mammalian dung in an underground cavity, laying a single egg on each brood ball. A brood ball is the whole food resource for a larva to adulthood (Halffter 1997). Because sex in Scarabaeidae, including dung beetles, is determined by random allocation of XY sex chromosomes to offspring (Yadav et al. 1979), beetle parents do not know their offspring sex. In fact, brood ball size does not differ between sons and daughters in all of *Copris acutidens*, *Onthophagus ater*, *O. atripennis*, *O. fodiens* (Kishi unpublished data). For example, in *O. atripennis*, the average brood ball size for a son 1.45 ± 0.049 g (average ± standard error, *N* = 31) is not different from that for a daughter 1.42 ± 0.095 g (*N* = 32) (Student’s *t*-test, *t* = 0.35, df = 61, *P* = 0.73) (data from control treatment in Kishi and Nishida 2008). Horns of male dung beetles are used for male–male combat and are considered to be a result of sexual selection on males (Emlen et al. 2005). However, larger males generally win and thus achieve greater reproductive success than smaller males, although smaller males sometimes succeed in mating with a female guarded by a larger male (Hunt and Simmons 2000). In this study, we compared sexual shape dimorphism and SSD between Japanese dung beetles and other horned beetles in the same family, Scarabaeidae. We measured several morphological traits of Japanese dung beetles, but got out those data of other horned beetles from a data list reported by Kawano (2006).

We measured adult head width and length of horns or horn-like traits in seven common species of Japanese dung beetle: *Onthophagus ater*, *O. fodiens*, *O. atripennis*, *O. lenzii*, *O. ohbayashii*, *Caccobius unicornis*, and *Copris acutidens*. The beetles were collected in baited traps at Kyoto and Nara, Japan, in 2002 (Kishi and Nishida 2008). We randomly picked up 20 males and 20 females of each species for measurement. Under a stereomicroscope, head width was measured at its widest width in dorsal view, and horn length was measured as the straight-line distance from the base of the horn to its tip (Kawano 2006). For males of *O. ater*, *O. fodiens* and *O. lenzii*, the mean length of two horn-like exaggerated traits on pronotum was measured. For males of *O. atripennis*, the mean length of two head horns was measured. For males of *C. unicornis* and *C. acutidens*, the length of a single head horn was measured. For males of *O. ohbayashii*, the mean length of tibiae, which develop quite longer in males, was measured. The degree of SSD was defined as the mean head width of males divided by that of females. The degree of sexual shape dimorphism was evaluated by calculating the allometric index *α* (Kawano 2006). In this index, it is assumed that sexually dimorphic traits more rapidly grow as other sexual monomorphic traits grow among individuals.

At first, we tested whether a group of SSD values in the dung beetles was different from that of a mother population with a mean value 1.0 and a standard normal distribution by using one-sample *t*-test. We similarly tested a group of SSD values at horned beetles. Then, we compared the mean SSD value of dung beetles with that of horned beetles by using the nonparametric Wilcoxon test. To examine that the degree of sexual shape dimorphism significantly differs between the two groups, we performed the Wilcoxon test to compare *α* values between the two groups. All statistical analyses were carried out by using R version 3.1.1 software (R Development Core Team 2014). No parental provisioning after oviposition is known in the 19 horned beetle species (Kawano 2006).

## Results

### Model simulations

**Figure 2:**
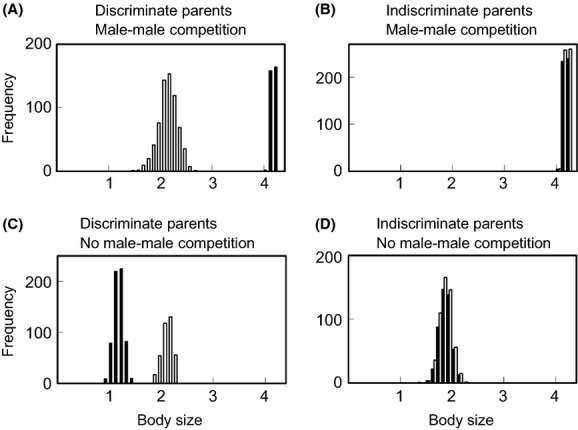
Frequency distributions of body size in the final populations, realized at the evolutionarily stable state after 1000 generations when parents distinguish (A, C) or do not distinguish (B, D) the sex of their offspring, and when male–male competition exists (A, B) or does not (C, D). Open and filled bars indicate females and males, respectively.

Similar results were observed in 100 simulation runs of each model, in which parents distinguished the sex of offspring or not, and male–male competition occurred or not. In the models DC and Dc, with the parents’ distinction of offspring sex, SSD evolved and sons and daughters had different optimal sizes (Fig.2A and C), while SSD did not evolve and sons and daughters had a similar body size in the models dC and dc (Fig.2B and D). In the model dC, remarkably, the peak frequency of both sexes reached to the optimal size for a son, but not settled at the intermediate size between the female optimal size and the male optimal size (Fig.2B). In the model dc, furthermore, the peak frequency of both sexes was at the optimal size for a daughter around 2.0 (Fig.2D).

**Figure 3:**
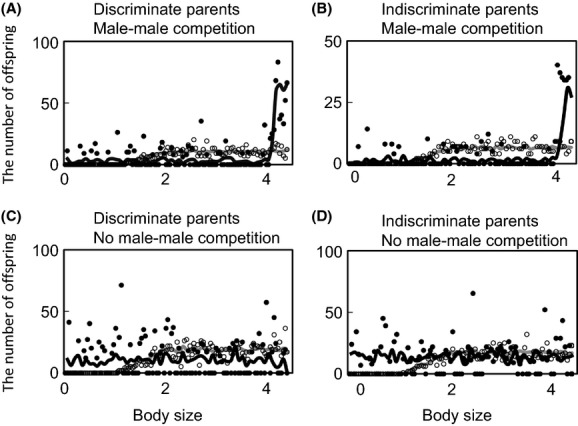
The number of offspring left by an individual of a given size, obtained from simulations when parents do (A, C) or do not (B, D) distinguish the sex of offspring in the phase of resource allocation, and when male–male competition does (A, B) or does not (C, D) exist. Open and closed circles indicate females and males, respectively, and each of them represented by 100 of 3000 individuals for simplicity. The gray and black lines are smoothed moving averages of data for females and males, respectively.

Size–fitness curves of the models DC and dC show that only largest males attained greater reproductive success, resulting in greater variance in reproductive success in males than in females (Fig.3A and B). In models Dc and dc, meanwhile, size–fitness curves of males resulted in little variance in reproductive success (Fig.3C and D). Size–fitness curves of females were similar among the four models (Fig.3). The expected fitness returns for sex-indiscriminate parents were the means of the reproductive success of a son and that of a daughter for a given body size. Then, when males competed for mates (model DC and dC), the expected fitness return for sex-indiscriminate parents ascended sharply at around the optimal size for a son (i.e., 4.0), indicating that the offspring size converged to the optimal size for a son (Fig.3B). When males did not compete for mates (model Dc and dc), size–fitness curves of males crossed those of females at around the optimal size for a daughter (i.e., 2.0) (Fig.3C and D). Then, the expected fitness return for parents in the models dC and dc ascended at the optimal body size for a daughter, indicating that the offspring size also converged to the optimal size for a daughter (Fig.3D).

### Specimen measurements

**Figure 4:**
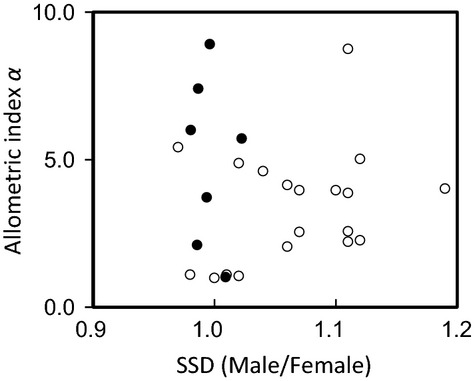
Sexual size dimorphism (SSD) values are close to 1.0 in Japanese dung beetles (closed circles), unlike in other scarabaeid horned beetles (open circles), whereas the variance in the allometric index *α*, an indicator of sexual dimorphism, does not differ between the two groups.

In seven dung beetle species, the average SSD (male/female ratio of pronotum width) was 1.007 ± 0.004 (mean ± SE), virtually equivalent to unity (*t* = 1.25, df = 6, *P *= 0.26, Fig.4). In the 19 horned beetle species reported by Kawano (2006), the average SSD was 1.067 ± 0.013, significantly different from that of the mother population (*t* = 5.08, df = 18, *P* < 0.0001) and significantly larger than that of the dung beetles (*χ*^2^ = 6.04, df = 1, *P* = 0.014, Fig.4). In contrast, the allometric index *α* varied over a similar range in both groups (Wilcoxon test, *χ*^2^ = 1.35, df = 1, *P* = 0.25, Fig.4). Thus, SSD was significantly more limited in the Japanese dung beetles than in the horned beetles, whereas the degree of sexual shape dimorphism varied similarly in both groups.

## Discussion

Results of both approaches showed that crucial and sex-indiscriminate parental provisioning prevents SSD, but not the evolution of sexual shape dimorphism. Simulation results showed that the evolutionarily stable provisioning way of the sex-indiscriminate parents is to provide the optimal amount of food for a son to each of both sons and daughters when males compete for mates. Then, sons receive enough amount of food to develop their body size and sexual ornaments, and daughters receive excessive amount of food more than the optimal amount which maximizes the fitness return for parents per investment. This excessive investment for daughters can be explained by two fitness curves of a son and a daughter (Fig.3A and B). If parents diminish the amount of food per offspring, their sons in future are likely to lose in male–male combat and then attain little reproductive success. Then, to maximize the fitness return, parents have to put priority on providing each son with the optimal amount of food, even if providing the wasteful food for daughters. It means that males always receive enough food resources to develop weapon and body size, and then, crucial parental provisioning does not prevent the evolution of sexual shape dimorphism. On the other hand, when male–male competition does not occur, the optimal amount of food for a daughter becomes larger than that for a son. In this situation, sex-indiscriminate parents in turn evolved to provide the optimal amount of food for a daughter to each offspring of both sexes. Then, whichever the optimal amount of food for a son or for daughter is larger, sex-indiscriminate parents evolved to provide the larger one of the two optimums to each of both sons and daughters. This means that even when parents do not know the sex of offspring, the evolutionarily stable amount of food for a single offspring always evolves to be identical to the larger one of the two optimums.

Empirical data of dung beetles and other horned beetles fully supported the simulation results. In Japanese dung beetles, SSD was significantly rarer than other horned beetles, but the degree of sexual shape dimorphism did not differ between those two beetle groups. It indicates that even combination of crucial and sex-indiscriminate parental provisioning does not prevent the evolution of sexual shape dimorphism, but it limits SSD in dung beetles. To demonstrate this conclusion more clearly, leaf-rolling beetles in Attelabidae and related Rhynchitidae may be another testable taxon, because various patterns of parental provisioning and male–male competition have been known (Kobayashi et al. 2012).

This study may give an important implication that the realized body size may not be optimal, rather excessively larger in one sex, of which the optimal amount of food is smaller, when parents feed both sexes of offspring indiscriminately. Even in mammals and birds, the observed amount of parental provisioning is not different between two sexes of offspring, but rather depends on other, more sensitive factors, such as environmental variability and hatching order (Blanckenhorn 2005), except for some mammals and birds that distinguish between sexes of offspring (Clutton-Brock 1991). Therefore, in most mammals, birds and even other organisms with parental provisioning, the degree of SSD may be an inappropriate index to measure the strength of sexual selection. Rather, the degree of sexual shape dimorphism may be the better index when parents do not distinguish the sex of offspring, although many other selective forces may also affect the evolution of sexual shape dimorphism (Dunn et al. 2001).

This study can offer another possible implication to the evolution of parental investment pattern, provisioning offspring one by one or several brood mates together. Results of fitness curves indicate that the reduced amount of food for a son results in the steep reduction of fitness return for parents due to male–male competition. Then, in nature dung beetle, parents should minimize variance of the food amount among their offspring. Because within-brood competition is a major causal mechanism of food variation among offspring (Wright and Leonard 2002), natural and sexual selection may favor parents that provision offspring one by one to avoid within-brood competition when male–male competition occurs. In fact, parents of horned dung beetles provision offspring one by one, producing a brood ball for each offspring (Halffter 1997), while parents of some burying beetles with no male–male combat feed their larvae together (Eggert and Muller 1997; Scott 1998). At last, we remark that the sex-indiscriminate parental investment between offspring sexes should be investigated by more researches, as with the sex-discriminate parental investment.

## Supporting Information

**Appendix S1.** Degrees of sexual size dimorphism (male/female) and sexual dimorphism in Japanese dung beetles and other Scarabaeidae beetles reported by Kawano (2006).
