# Supplementary material for: Sexual shape dimorphism accelerated by male–male competition, but not prevented by sex-indiscriminate parental care in dung beetles (Scarabaeidae)
Source: Ecol Evol. 2015 Jun 19;5(14):2754–61. doi: 10.1002/ece3.1558 (PMC4541983; doi:10.1002/ece3.1558)
Supplement: Supplementary file 1 [file ece30005-2754-sd1.docx]

Appendix. Degrees of sexual size dimorphism (male/female) and sexual dimorphism in Japanese dung beetles and other Scarabaeidae beetles reported by Kawano (2006).

| Kawano (2006) | SSD | Allometric index | Japanese dung beetles | SSD | Allometric index |
| --- | --- | --- | --- | --- | --- |
|  | in body length | *α* |  | in head width | *α* |
| Monomorphic | | | | | |
| *Protaetia brevitarsis* Lewis | 0.98 | 1.10 | *Phelotrupes auratus* Motschulsky | 1.01 | 1.02 |
| *Protaetia lenzi* Harold | 1.02 | 1.06 |  |  |  |
| *Protaetia orientalis* Goly et Percheron | 1.01 | 1.10 |  |  |  |
| *Rhomborrhina japonica* Hope | 1.00 | 0.99 |  |  |  |
| Dimorphic | | | | | |
| *Allomyrina dichotoma* L. | 1.11 | 2.57 | *Caccobius unicornis* Fabricius | 1.00 | 8.91 |
| *Augosoma centaurus* Fabricius | 0.97 | 5.42 | *Copris acutidens* Motschulsky | 1.02 | 5.71 |
| *Chalcosoma atlas* L. | 1.07 | 3.96 | *Onthophagus atripennis* Waterhouse | 0.99 | 3.72 |
| *Chalcosoma caucasus* Fabricius | 1.19 | 4.02 | *Onthophagus fodiens* Waterhouse | 0.99 | 7.40 |
| *Chalcosoma moellenkampi* Kolbe | 1.11 | 3.87 | *Onthophagus lenzii* Harold | 0.98 | 6.00 |
| *Dynastes hercules* L. | 1.10 | 3.96 | *Onthophagus ohbayashii* Nomura | 0.99 | 2.11 |
| *Dynastes neptunus* Quansel | 1.11 | 8.75 |  |  |  |
| *Eupatorus gracilicornis* Arrow | 1.12 | 5.02 |  |  |  |
| *Eupatorus siamensis* Castelnau | 1.04 | 4.61 |  |  |  |
| *Megasoma janus* Felsche | 1.07 | 2.55 |  |  |  |
| *Pachyoryctes solidus* Arrow | 1.02 | 4.88 |  |  |  |
| *Xylotrupes gideon* L. | 1.06 | 4.14 |  |  |  |
| *Cheirotonus gestroi* Pouillaude | 1.12 | 2.27 |  |  |  |
| *Cheirotonus parryi* Gray | 1.11 | 2.22 |  |  |  |
| *Euchirus dupontianus* Burmeister | 1.06 | 2.05 |  |  |  |
